# Evaluation of Graphene as a Novel Bioactive Stent Coating: Comparative Performance and Vascular Response in Porcine Coronary Arteries

**DOI:** 10.3390/jfb17070313

**Published:** 2026-06-28

**Authors:** Jacek Arkowski, Przemysław Sareło, Urszula Pasławska, Robert Pasławski, Magdalena Wawrzyńska

**Affiliations:** 1 Pre-Clinical Research Center, Wrocław Medical University, K. Marcinkowskiego 1, 50-368 Wrocław, Poland; jacek.arkowski@umw.edu.pl (J.A.); magdalena.wawrzynska@umw.edu.pl (M.W.); 2 Department of Biomedical Engineering, Faculty of Fundamental Problems of Technology, Wrocław University of Science and Technology, Wybrzeże S. Wyspiańskiego 27, 50-370 Wrocław, Poland; 3 Department of Diagnostics and Clinical Sciences, Faculty of Veterinary Medicine, University of Agriculture in Kraków, Aleja A. Mickiewicza 21, 31-120 Kraków, Poland; urszula.paslawska@urk.edu.pl (U.P.); robert.paslawski@urk.edu.pl (R.P.)

**Keywords:** graphene-coated stents, cardiovascular biomaterials, vascular healing, endothelialization, optical coherence tomography, sirolimus-eluting stents, swine model, surface modification, in-stent restenosis

## Abstract

Coronary drug-eluting stents (DESs) are the current clinical standard, yet delayed endothelialization remains a critical challenge. Graphene-based coatings have emerged as promising cardiovascular biomaterials due to their favorable hemocompatibility and ability to support endothelial cell growth. In this study, we evaluated the in vivo performance of graphene-coated stents (GCSs) compared with commercial sirolimus-eluting stents in a Polish White swine model (*n* = 10). Stents were implanted into major coronary branches, with follow-up at 30 and 90 days using quantitative coronary angiography (QCA), optical coherence tomography (OCT), and cryogenic scanning electron microscopy (cryo-SEM). No systemic toxicity, mortality, thrombotic events, or ischemic complications were observed during the study period. QCA demonstrated no significant differences in percent diameter stenosis between GCSs and DESs at either 30 days (12.3 ± 6.1% vs. 8.6 ± 5.8%, *p* = 0.2782) or 90 days (18.3 ± 10.5% vs. 9.6 ± 6.6%, *p* = 0.1074). OCT analysis confirmed comparable lumen and neointimal parameters between groups, while demonstrating a favorable, although non-significant, trend toward a lower percentage of uncovered struts in GCSs. Cryo-SEM imaging demonstrated stable tissue integration and a preserved healing response surrounding GCSs. Collectively, these findings indicate that GCSs are safe and biocompatible and demonstrate mid-term vascular performance comparable to clinically used DES platforms. The presented results support further investigation of graphene-based coatings as potential surface-modification strategies for coronary stents.

## 1. Introduction

Percutaneous coronary intervention (PCI) with stent implantation remains one of the most performed procedures in contemporary cardiovascular medicine and constitutes a cornerstone of coronary artery disease (CAD) treatment. Over the past decades, interventional cardiology has evolved from plain balloon angioplasty toward increasingly advanced stent technologies aimed at improving vascular patency and long-term clinical outcomes [1]. Despite continuous technological progress, CAD remains one of the leading causes of morbidity and mortality worldwide, emphasizing the persistent need for further optimization of coronary implant design and vascular healing strategies [2].

The introduction of drug-eluting stents (DESs) markedly reduced restenosis rates compared with bare-metal stents (BMSs). Nevertheless, several clinically relevant limitations associated with currently used DES platforms remain unresolved, including delayed endothelial healing, chronic vascular inflammation, late thrombotic complications, hypersensitivity reactions related to polymer coatings, and prolonged requirements for dual antiplatelet therapy [3]. Consequently, contemporary cardiovascular biomaterials research increasingly focuses on the development of pro-healing surface modifications capable of promoting endothelial recovery while simultaneously limiting thrombogenicity and excessive neointimal hyperplasia [4,5,6].

Current approaches to stent surface engineering extend beyond classical antiproliferative drug-delivery systems and include physicochemical surface modifications designed to improve hemocompatibility, reduce platelet activation, and modulate vascular cell behavior [7,8,9]. Additional strategies involve the incorporation of bioactive molecules, including antibodies, cytokines, and endothelial progenitor cell-capturing agents intended to accelerate re-endothelialization and attenuate inflammatory responses following implantation [10,11,12,13]. Despite these advancements, there remains a demand for novel biomaterial coatings capable of simultaneously supporting vascular healing, reducing inflammatory activation, and maintaining long-term mechanical stability.

Among emerging cardiovascular biomaterials, graphene and its derivatives, including graphene oxide (GO) and reduced graphene oxide (rGO), have attracted substantial attention as potential surface-modification materials for implantable medical devices and coronary stents [14]. Graphene-based materials exhibit unique physicochemical properties, including high mechanical strength, nanoscale surface topography, electrical conductivity, high specific surface area, and favorable protein adsorption characteristics. These features may facilitate endothelial cell adhesion and proliferation while reducing platelet activation, inflammatory signaling, and thrombogenicity [15,16,17,18]. Previous in vitro studies further demonstrated that graphene-coated surfaces may simultaneously promote endothelialization and limit smooth muscle cell proliferation and inflammatory activation [19,20,21]. In addition, graphene-containing stent coatings demonstrated improved hemocompatibility, reduced platelet adhesion, and enhanced endothelial cell growth while preserving the desirable mechanical properties of the underlying metallic platform [15,17].

Our previous investigations demonstrated successful deposition of graphene-based coatings onto cobalt–chromium coronary stents, resulting in enhanced endothelial cell proliferation, preserved biocompatibility, and favorable mechanical characteristics (including stable graphene layer after bench deployment) under experimental conditions [21]. However, despite promising in vitro findings and preliminary small-animal investigations, there remains a substantial lack of large-animal in vivo studies directly comparing graphene-coated coronary stents (GCSs) with clinically approved DES platforms using clinically relevant intravascular imaging modalities. Large-animal coronary models combined with advanced intravascular imaging techniques are considered essential for translational evaluation of novel cardiovascular biomaterials prior to potential clinical application.

Therefore, the aim of the present study was to comparatively evaluate the vascular response and mid-term safety profile of GCSs and commercially available sirolimus-eluting stents in a swine coronary artery model. The assessment was performed at 30- and 90-day follow-up using quantitative coronary angiography (QCA), optical coherence tomography (OCT), clinical monitoring, and ex vivo cryogenic scanning electron microscopy (cryo-SEM). The presented results provide translational preclinical insights into the feasibility, vascular compatibility, and healing characteristics of graphene-based coronary stent coatings.

## 2. Materials and Methods

### 2.1. Animal Facilities and Welfare with Pre- and Post-Implantation Monitoring

The investigation was conducted using an in vivo swine model at the Institute of Veterinary Medicine, Nicolaus Copernicus University in Toruń, Poland. All procedures were approved by the Local Ethics Committee for Animal Experiments (approval no. 11/2023) and were performed in full compliance with applicable Polish and European Union regulations concerning the care and use of laboratory animals.

A total of 10 Polish White Swine (*Sus scrofa domestica*) were included in the study, divided into two cohorts of 5 animals each, based on the follow-up duration: a 30-day (1-month) observation group and a 90-day (3-month) observation group. At the time of study initiation, animals weighed approximately 35 kg and were determined to be in good health. Animals were housed in species-appropriate conditions with controlled temperature, humidity, and a 12 h light/dark cycle. They received a standard pig diet and had ad libitum access to water throughout the study. Comprehensive clinical evaluations were performed at multiple time points (at day 0, day 30, and day 90) to monitor the general health status and cardiac function of the animals. These included physical examination, hematological and biochemical blood tests, 12-lead electrocardiogram (ECG), and coronary angiography. All procedures were performed under general anesthesia using a combination of propofol, fentanyl, and isoflurane. The animals were intubated and mechanically ventilated. At the end of the study, the animals were subjected to euthanasia.

### 2.2. The Procedure of Stent Implantation in Swine Main Coronary Artery Branches

Vital parameters of the animals, including continuous ECG, were always monitored throughout the stent implantation procedure to ensure animal safety and procedural stability. Transthoracic cardiac echocardiography was performed at three points: prior to stent implantation (day 0), immediately following the procedure (day 0), as well as at follow-up on either day 30 or day 90, depending on the assigned animal cohort. Echocardiographic assessments included measurement of left ventricular ejection fraction (LVEF), evaluation of regional contractility, and assessment of heart valve function.

Once the intravenous canulae were inserted into ear veins, the sedation and anesthesia were induced. The intravenous access was maintained until the end of the procedure. Then, the femoral artery was punctured, and a guidewire and a vascular sheath was inserted. When the coronary artery ostium was engaged with a guiding catheter, a contrast medium (Iomeron, Bracco Imaging Deutschland GmbH, Konstanz, Germany) was injected to obtain coronary angiogram. The stent implantation system was inserted into the coronary artery (e.g., guidewire, balloon catheter with a stent). The stents were mostly implanted into three main coronary artery branches in each animal (left anterior descending—LAD, left circumflex coronary artery—LCx and right coronary artery—RCA). Study stents were randomly allocated to two main branches in each animal and the control stent was implanted in the remaining branch. Three stents were successfully implanted as intended in eight animals, while in two remaining animals only two stents (one study stent and one control stent) were implanted. The study stent was a 3 × 15 mm cobalt–chrome platform coated with a graphene layer. The control stent was a commercially available cobalt–chrome sirolimus eluting Alex Plus (Balton sp. z o. o., Warsaw, Poland) stent. Of note, both stents (the study stent and the DES used as the control) are built on the same cobalt–chrome platform. Thus, any difference in stent performance should be attributed to the coating and not to the platform material and/or construction.

After stent implantation the vascular sheath was removed from the femoral artery, and the puncture site was compressed to prevent bleeding. The anesthesia gases were stopped, and the venous canulae, intratracheal tube, and ECG electrodes were removed. Just before canula removal, an analgesic was given intravenously, and a long-acting antibiotic was administered intramuscularly. The animals were transferred into pens for the observation period (30 and 90 days).

### 2.3. The Coronary Angiography with QCA

A JR4 6F guiding catheter was used to selectively engage coronary ostium. The contrast medium injection was recorded in two orthogonal views (e.g., right anterior oblique at 30° and left anterior oblique at 60°). The coronary angiography was carried out at the following timepoints: day 0—directly after stent implantation (baseline), day 30—short term observation cohort, and day 90—medium term observation cohort.

In the QCA analysis, the following parameters were measured: MLD—minimal lumen diameter, RD—reference diameter, %DS—percent of diameter stenosis, AG—acute gain, LL—late lumen loss.

### 2.4. The Analysis of the Stented Segment by Means of OCT

Following an intravenous bolus administration of 5000 units of unfractionated heparin, intravascular imaging was performed using the Dragonfly OpStar™ Imaging Catheter (Abbott Medical sp. z o. o., Warsaw, Poland). The catheter was advanced over an angioplasty guidewire to a position approximately 20 mm distal to the implanted stent. An automated pullback was initiated concurrently with contrast medium injection to ensure optimal blood clearance and image acquisition.

In cases where the initial imaging was suboptimal—typically due to inadequate engagement of the coronary ostium or mixing of blood and contrast—the catheter was repositioned, and the imaging sequence was repeated until satisfactory image quality was achieved.

Transverse cross-sectional images of the stented artery segments were acquired and analyzed using automated contour tracing software. The reference vessel dimensions were derived from measurements taken 5 mm proximal and 5 mm distal to the stent edges. The proximal and distal boundaries of the stented segment were defined as the first and last cross-sections where stent struts were clearly visible in at least 50% of the vessel circumference.

Cross-sectional images were analyzed at 1 mm intervals (corresponding to every 10 image frames). If a specific cross-section was deemed unsuitable for analysis due to vessel curvature, side branches, poor image quality, or artifacts (e.g., reverberations), the closest adjacent analyzable section was selected.

The stent area was delineated by tracing the outer edge of the visible stent struts, while the vessel lumen area was defined by tracing the inner edge of the struts or overlying neointimal tissue. In cases of malapposed stent struts, the stent contour was defined by the outer edge of the unapposed struts. Struts were categorized as well-apposed or malapposed, with malapposition defined as a gap of greater than 100 μm between the stent strut and the vessel wall. Additionally, each strut was evaluated for neointimal coverage and classified as either covered or uncovered. The neointimal area was calculated as the difference between the stent area and the lumen area.

A total of 15 cross-sections per stent were analyzed, yielding 255 cross-sections for the graphene-coated stents and 150 cross-sections for the commercially available drug-eluting stents. Quantitative OCT parameters—vessel lumen area, stent area, and neointimal area—were first averaged for all analyzed cross-sections within each individual stent. Subsequently, statistical comparisons between the study groups were performed using per-stent mean values, with each implanted stent considered an independent experimental unit. The neointimal cross-sectional area was specifically defined as neointimal area=stent area−lumen area.

### 2.5. The Ex Vivo Stent Surface Visualization by Cryo-SEM

After euthanasia, hearts were excised immediately and rinsed with physiological saline to remove residual blood. Each heart was then immersed in 10% neutral-buffered formalin for fixation. Following adequate fixation, coronary arteries containing the implanted study and control stents were carefully dissected from the surrounding myocardium. The vessels were longitudinally opened, and the stents were gently removed to preserve the luminal surface and any adherent biological material. Ex vivo stent surface imaging was performed using a scanning electron microscope AURIGA 60 (Carl Zeiss Microscopy GmbH, Oberkochen, Germany). For cryo-analysis, samples were processed in a cryo-SEM preparation chamber with an integrated transfer system mounted on the platform, enabling rapid freezing, fracturing, sublimation, and subsequent low-temperature imaging. Imaging parameters, including accelerating voltage, working distance, and detector configuration, were optimized individually for each modality to achieve high-resolution visualization of stent strut morphology and biological deposits.

### 2.6. Statistical Analysis

The statistical analysis of the obtained data sets was performed in GraphPad Prism 11 (Dotmatics, Boston, MA, USA). The Shapiro–Wilk test was used to assess normality and the Levene’s test was used to assess homogeneity of variance. The significance level was set to 0.05. The data with normal distribution were compared with unpaired *t* test; otherwise, the Mann–Whitney test was used with the 0.05 significance level.

## 3. Results

### 3.1. Clinical and Systemic Evaluation Confirmed Absence of Adverse Events or Abnormalities

Throughout the 30-day and 90-day observation periods, no clinical adverse events were recorded in any of the animals. All swine maintained normal activity levels, appetite, and behavior. There were no noted deaths during the study, and all animals remained in good general condition until the end of their respective observation periods.

Hematological and serum biochemical analyses conducted at baseline and at the designated follow-up timepoints revealed no significant abnormalities (Table 1). Parameters including red blood cell count (RBC), hemoglobin (Hb), hematocrit (HCT), and white blood cell count (WBC) remained within physiological ranges. Similarly, serum urea, creatinine, hepatic enzymes (aspartate aminotransferase—AST and alanine aminotransferase—ALT), and cardiac troponin I levels did not indicate renal, hepatic, or myocardial injury at either follow-up point.

ECG analysis demonstrated no signs of arrhythmia, ischemia, or other adverse electrical activity. Sinus rhythm was preserved in all animals, with stable heart rate and normal conduction intervals at both the time of stent implantation and at follow-up (Table 2). No ST-segment deviations or abnormal T-wave morphology were observed.

Transthoracic echocardiography confirmed preserved global cardiac function at all time points. LVEF remained within normal limits, and no segmental wall motion abnormalities were detected. The valve structure and function were also unremarkable, and no intracardiac flow disturbances were noted (Table 3).

Finally, macroscopic evaluation at necropsy revealed no pathological changes in any of the major organs in both the 30-day and 90-day cohorts.

### 3.2. QCA Analysis Shows Consistent Vascular Outcomes Across Study Duration

**Figure 1:**
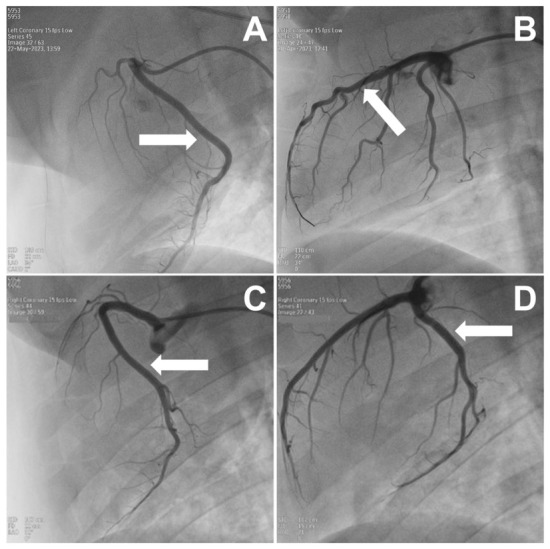
Representative angiographic images of QCA analysis at 30 days post-implantation, where control stent was implanted in RCA (**A**) and study stent was implanted in LAD (**B**). Similarly, representative angiographic images at 90 days post-implantation, where control stent was implanted in RCA (**C**) and study stent was implanted in LCx (**D**). The locations of the implanted stents in the coronary arteries are indicated by arrows.

**Figure 2:**
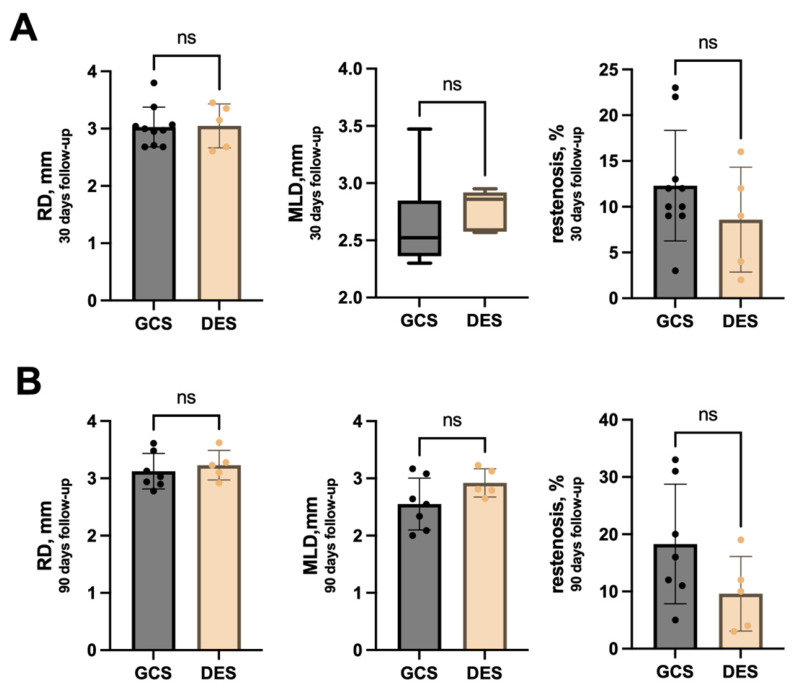
Graphs comparing the mean values (or median, depending on the assumption of normality of distribution based on the Shapiro–Wilk test) with the standard deviation (or minimum and maximum values) of the parameters reference diameter (RD), minimal lumen diameter (MLD) and percentage of restenosis for the study stents (GCS) and control stents (DES) for two time points: 30 days (panel **A**) and 90 days (panel **B**). The comparison was performed using the unpaired *t* test or the Mann–Whitney test at the statistical significance level of 0.05. ns—nonsignificant.

At 30 days post-implantation, QCA analysis revealed no statistically significant adverse effects, including in-stent stenosis. There were also no significant differences in lumen diameter between the study stent and the control stent. Similarly, at 90 days, no significant in-stent stenosis or other adverse changes were observed. Lumen measurements remained comparable between the study and control stents, with no statistically significant differences noted (Table 4, Figure 1 and Figure 2).

### 3.3. Intravascular OCT Confirms the Comparable Performance of the Study Stent to Clinically Used Ones

OCT imaging was performed at the stent cross-section level, generating virtual histology of the vessel wall and the implanted stents. This allowed detailed visualization of the vessel wall structures, including the intima and media. No significant lesions were detected in either layer at any timepoint, and no signs of adverse phenomena such as intravascular thrombosis at the stent struts were observed.

**Figure 3:**
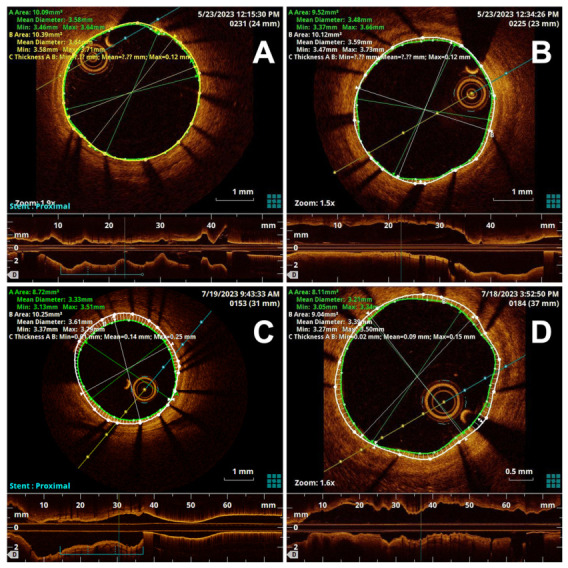
Representative images obtained by means of OCT and parameters analysis at 30 days post-implantation for control stent (**A**) and study stent (**B**). Similarly, representative images at 90 days post-implantation for control stent (**C**) and study stent (**D**).

**Figure 4:**
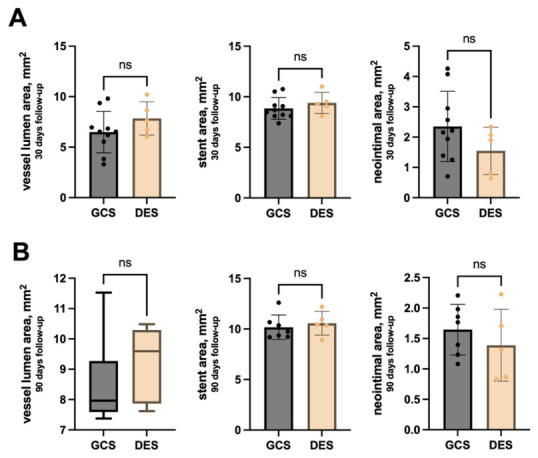
Graphs comparing the mean values (or median, depending on the assumption of normality of distribution based on the Shapiro–Wilk test) with the standard deviation (or minimum and maximum values) of the parameters lumen area, stent area and neointimal area for the study stents (GCS) and control stents (DES) for two time points: 30 days (panel **A**) and 90 days (panel **B**). The comparison was performed using the unpaired t test or the Mann–Whitney test at the statistical significance level of 0.05. ns—nonsignificant.

At 30 days post-implantation (Table 5, Figure 3 and Figure 4), there were no statistically significant differences between the study and control stents in key parameters, including mean lumen cross-sectional area, neointimal area, and the percentage of uncovered stent struts. However, a trend toward a higher number of covered stent struts was observed in the study stents compared to the control group.

At 90 days (Table 5, Figure 2 and Figure 3), OCT analysis continued to show no significant differences between the study and control stents with respect to mean lumen area, stent cross-sectional area, neointimal area, or percentage of uncovered struts.

### 3.4. Ex Vivo Cryo-SEM Imaging Confirms Favorable Tissue Integration of Graphene-Coated Stents

Ex vivo cryo-SEM imaging provided detailed insight into the biological integration of both the control (i.e., DES) and study (i.e., GCS) stents. The cryogenic preparation preserved the native architecture of tissues adherent to the stent struts, enabling visualization of vascular healing responses and tissue–stent interaction.

**Figure 5:**
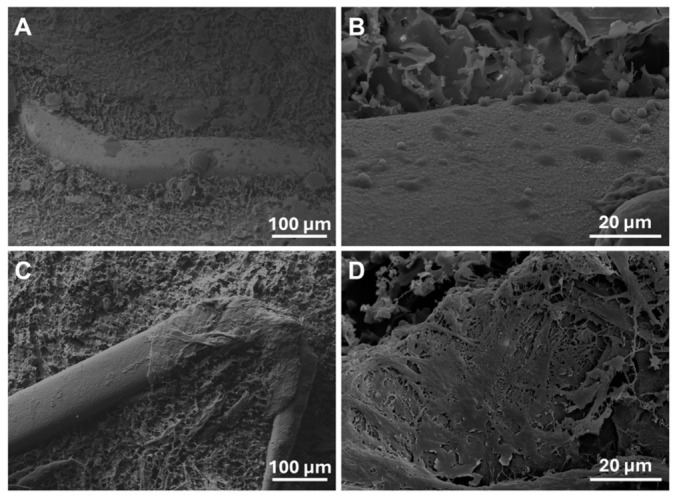
Representative images obtained by means of cryo-SEM. Panels (**A**,**B**) show control stent (DES) surfaces at 90 days post-implantation, with (**B**) presented at higher magnification. Similarly, panels (**C**,**D**) show study stent (GCS) surfaces at 90 days post-implantation, with (**D**) presented at higher magnification. Scale bar is presented at 20 μm and 100 μm.

Across all examined samples, both the DES and the GCS stents were embedded within the vessel wall and demonstrated tissue coverage of the stent struts, indicating successful vascular integration and neointimal formation. No qualitative evidence of thrombus formation, abnormal tissue reactions, excessive neointimal overgrowth, or incomplete strut coverage was observed in either group (Figure 5).

Qualitative differences in tissue organization were observed between individual samples. However, these findings were not quantified and therefore should be interpreted cautiously. The cryo-SEM observations indicate that GCSs exhibit vascular compatibility and healing characteristics comparable to those of the reference DESs.

Overall, cryo-SEM findings support the favorable biocompatibility of the graphene-coated platform and confirm successful incorporation into the vessel wall without evidence of adverse healing responses during the study period.

## 4. Discussion

Despite substantial advances in interventional cardiology, the biological response to coronary stent implantation remains a major determinant of long-term procedural success. Although stent implantation effectively restores vessel patency, the presence of a permanent foreign material within the highly biologically active vascular wall initiates a cascade of inflammatory and thrombotic reactions. Rapid restoration of an intact endothelial layer is considered one of the key factors limiting thrombosis and maintaining long-term vascular compatibility. Conversely, excessive neointimal hyperplasia may result in restenosis and recurrent vessel narrowing, thereby reducing the long-term efficacy of the intervention [22].

Over the past three decades, the development of coronary stents has focused on achieving a balance between inhibition of excessive smooth muscle cell proliferation and promotion of physiological endothelial healing. Contemporary DESs substantially reduced restenosis rates compared with BMS and currently demonstrate excellent clinical efficacy and safety profiles [23]. Nevertheless, delayed endothelialization, chronic inflammatory responses associated with polymer coatings, and the risk of thrombotic complications remain clinically relevant limitations. Moreover, successful DES implantation still requires prolonged dual antiplatelet therapy (DAPT), which may be problematic in patients with high bleeding risk, contraindications to prolonged antiplatelet treatment, or poor treatment compliance.

For these reasons, there is a continued interest in alternative stent materials and surface-modification strategies capable of improving vascular healing while maintaining mechanical stability and anti-restenotic efficacy [24]. Several next-generation cardiovascular implants, including bioresorbable magnesium-based and polymer-based scaffolds, have reached clinical evaluation. Although their bioresorbable nature initially appeared promising, important limitations such as insufficient radial strength and inflammatory reactions associated with scaffold degradation remain unresolved [25,26].

Alternative interventional strategies, such as drug-eluting balloons, have also emerged in recent years. While these technologies avoid the implantation of a permanent metallic scaffold, current evidence suggests that in most clinical settings they provide outcomes comparable rather than superior to modern DES platforms. Nevertheless, they may offer advantages in selected cases, particularly in small vessels, complex lesions, or clinical scenarios in which prolonged DAPT is undesirable [27,28,29].

In parallel with these developments, considerable effort has been directed toward engineering bioactive stent coatings. Both organic coatings, including endothelial cells, antibodies, and cytokines, and inorganic coatings, such as oxides, carbides, nitrides, and carbon-based materials, have been investigated to improve vascular compatibility and endothelial recovery following implantation [30,31,32].

Among currently investigated biomaterials, graphene-based coatings appear particularly promising. Previous in vitro studies demonstrated that graphene may promote endothelial cell adhesion and proliferation while simultaneously reducing platelet activation and thrombogenicity. Favorable albumin-to-fibrinogen adsorption profiles observed on graphene-coated surfaces may further contribute to improved hemocompatibility and reduced thrombus formation [15]. In addition, graphene-based coatings demonstrated favorable mechanical properties and reduced platelet adhesion during in vitro testing, suggesting potential advantages for cardiovascular applications [17].

Several experimental studies have investigated graphene-containing composite coatings for cardiovascular implants. Graphene-gold coatings promoted endothelial cell growth on vascular implant surfaces [31]. Similarly, graphene-containing multifunctional coatings applied to magnesium alloys improved corrosion resistance while simultaneously enhancing endothelial compatibility and reducing platelet adhesion [33]. Other investigators demonstrated that functionalized graphene surfaces may facilitate nitric oxide-generating activity, potentially supporting vascular homeostasis and endothelial recovery following implantation [34].

In our previous study, we demonstrated successful deposition of graphene-based coatings onto cobalt–chromium coronary stents and confirmed their favorable mechanical and biological properties under experimental conditions [21]. GCSs supported significantly increased human umbilical vein endothelial cell proliferation compared with standard stent surfaces and demonstrated good biocompatibility without evidence of local or systemic toxicity. Moreover, the graphene-coated platforms exhibited preserved radial strength and favorable elastic properties [21].

The present study extends these observations into a clinically relevant large-animal coronary model. To the best of our knowledge, there are currently very limited in vivo studies evaluating GCS implanted into swine coronary arteries using clinically applicable intravascular imaging modalities. Previous investigations involving graphene oxide-containing multilayer coatings implanted into rabbit carotid arteries demonstrated favorable safety profiles and reduced restenosis rates [20]. However, differences in vascular anatomy, implantation sites, and experimental design limit direct comparison with coronary swine models.

In the present study, GCSs demonstrated favorable vascular compatibility and mid-term safety comparable to commercially available DESs. No thrombotic complications, systemic toxicity, ischemic changes, or significant adverse vascular responses were observed during follow-up. QCA and OCT analyses demonstrated comparable lumen dimensions, restenosis rates, and neointimal response between GCS and commercially available DES platforms. Importantly, OCT and cryo-SEM analyses demonstrated a vascular healing response comparable to that observed with the clinically used DES platform, without evidence of excessive neointimal growth or adverse tissue reactions. That may indicate a favorable vascular healing profile associated with graphene surface modification.

The present findings further support the feasibility of obtaining stable graphene-based coatings on clinically relevant coronary stent platforms. Importantly, the observed vascular response suggests that graphene coatings may provide biological compatibility comparable to currently used DESs while potentially supporting physiological endothelial recovery.

An additional consideration relates to the long-term biological fate of graphene-based materials. Although available experimental evidence suggests favorable biocompatibility and low toxicity of immobilized graphene coatings, data regarding their long-term persistence, degradation behavior, systemic biodistribution, and chronic biological effects remain limited. Extended preclinical studies incorporating toxicological, biodistribution, and molecular analyses will be necessary before clinical translation.

Another important consideration is the mechanical stability of graphene coatings during stent crimping, deployment, and long-term exposure to pulsatile vascular forces. Although previous mechanical testing of the investigated platform demonstrated preservation of coating integrity following expansion, the present study did not directly evaluate particulate shedding, coating delamination, or systemic biodistribution of potential graphene fragments [21]. Future investigations should incorporate dedicated coating durability testing, particle-release quantification, and biodistribution analyses.

Several limitations of the present study should be acknowledged. First, the study cohort was relatively small, and therefore it was underpowered to detect numerically low differences in outcomes. Second, the follow-up period was limited to three months and therefore long-term effects of GCS implantation remain unknown. Nevertheless, the study design was based on established preclinical methodologies for coronary stent evaluation in porcine models. The selected 30- and 90-day follow-up periods correspond to standard early and mid-term assessment intervals commonly used in translational coronary stent studies and are considered sufficient to identify acute and subacute complications following PCI, including thrombotic events, abnormal vascular healing, inflammatory reactions, and early neointimal responses [35,36,37]. Furthermore, each animal received both the investigational GCS and the control DES in separate coronary arteries, thereby reducing biological variability and increasing statistical efficiency. The sample size was determined using prospective power calculations and was consistent with previously published large-animal coronary stent investigations. Third, histopathological and molecular analyses of endothelialization and inflammatory signaling were not performed. Consequently, the mechanisms underlying the observed vascular response cannot be fully characterized. Furthermore, quantitative histopathological assessment was not performed. Therefore, endothelial coverage, neointimal maturity, inflammatory cell infiltration, and endothelial phenotype could not be directly evaluated. Future studies should incorporate histomorphometric analysis together with immunohistochemical markers such as CD31, CD34, and von Willebrand factor to provide mechanistic insight into vascular healing associated with GCSs. Moreover, while the lack of adverse events and normal autopsy findings suggest no clinically significant embolization of carbon-containing particles, no dedicated assessment of coating particle release, biodistribution, or potential long-term accumulation was performed. Finally, although the swine coronary model remains one of the most clinically relevant large-animal models for coronary interventions, preclinical findings do not necessarily translate directly into human clinical outcomes.

Nevertheless, the presented results provide important translational evidence supporting further investigation of graphene-based coatings for cardiovascular implants and justify future long-term studies incorporating histological and molecular assessment of vascular healing responses.

## 5. Conclusions

Graphene-coated coronary stents demonstrated favorable vascular compatibility and mid-term safety in a clinically relevant swine coronary artery model. Multimodal assessment using QCA, OCT, and cryogenic scanning electron microscopy confirmed stable vascular integration without evidence of thrombosis, excessive restenosis, systemic toxicity, or adverse vascular remodeling. The vascular response observed for graphene-coated stents was comparable to that of commercially available drug-eluting stents, while imaging findings suggested a tendency toward enhanced tissue coverage and favorable healing characteristics. These results support the feasibility of graphene-based surface modification for coronary stents and justify further long-term preclinical studies incorporating histopathological and molecular evaluation of endothelialization and inflammatory responses.

## Figures and Tables

**Table 1:** Hematological and serum biochemical parameters assessed on day 0 (day of stent implantation), day 30, and day 90, according to the assigned animal cohorts. The measured values remained within normal physiological ranges throughout the study period, and no significant abnormalities or deviations in systemic parameters were observed.

	Day 0	Day 30	Day 90
RBC, T∙L^–1^ (SD)	6.74 ± 0.26	6.74 ± 0.30	6.42 ± 0.27
Hb, g% (SD)	10.5 ± 0.3	10.4 ± 0.5	10.1 ± 0.6
HCT, % (SD)	37.3 ± 0.6	37.5 ± 0.5	34.9 ± 1.9
WBC, G∙L^–1^ (SD)	17.06 ± 0.80	16.80 ± 0.76	14.91 ± 0.16
Urea, mmol∙L^–1^ (SD)	3.0 ± 0.2	4.4 ± 0.2	3.6 ± 0.3
Creatinine, μmol∙L^–1^ (SD)	71.0 ± 2.3	122.2 ± 11.7	111.2 ± 11.1
AST, U∙L^–1^ (SD)	28.7 ± 1.6	33.4 ± 2.1	37.0 ± 3.9
ALT, U∙L^–1^ (SD)	63.7 ± 2.0	81.2 ± 3.8	119.8 ± 9.3
Troponin I, ng∙L^–1^ (SD)	26 ± 7	8 ± 3	6 ± 3

**Table 2:** ECG parameters recorded on day 0 (day of stent implantation), day 30, and day 90 of the observation period. Analysis of the ECG recordings revealed no significant abnormalities or deviations in cardiac electrical activity across all time points.

	Day 0	Day 30	Day 90
Heart rate, min^–1^ (SD)	88 ± 11	77 ± 6	84 ± 8
P, mV∙s^–1^ (SD)	0.13/0.04 ± 0.01/0.01	0.11/0.04 ± 0.01/0.01	0.11/0.04 ± 0.01/0.01
PQ, s (SD)	0.12 ± 0.01	0.12 ± 0.01	0.11 ± 0.01
QRS, s (SD)	0.06 ± 0.01	0.06 ± 0.01	0.06 ± 0.01
ST segment	Isoelectric	Isoelectric	isoelectric
T, mV∙s^–1^ (SD)	0.14/0.07 ± 0.01/0.01	0.14/0.07 ± 0.01/0.01	0.12/0.06 ± 0.01/0.01
QT, s (SD)	0.36 ± 0.02	0.38 ± 0.02	0.38 ± 0.01
Heart axis AQRS, ° (SD)	85.4 ± 7.0	94.4 ± 2.9	93.0 ± 4.4
rythm	sinus	sinus	sinus
Features of ischemia	non	non	non

**Table 3:** Echocardiographic parameters assessed on day 0 (at stent implantation), and in the 30-day and 90-day observation groups. Transthoracic echocardiography showed preserved cardiac function at all time points, with no abnormalities detected.

	Day 0	Day 30	Day 90
Heart rate, min^–1^ (SD)	89 ± 6	77 ± 6	80 ± 6
LA/Ao (SD)	1.36 ± 0.07	1.39 ± 0.03	1.40 ± 0.02
RVDd, mm	16.9 ± 1.0	15.6 ± 0.9	19.2 ± 1.7
IVSd, mm	9.1 ± 0.8	9.4 ± 0.6	13.0 ± 0.8
IVSs, mm	12.2 ± 1.0	13.4 ± 0.9	15.6 ± 0.6
LVDd, mm	40.2 ± 2.0	41.0 ± 1.5	39.4 ± 1.7
LVDs, mm	28.0 ± 1.2	25.1 ± 0.7	25.8 ± 0.9
LWDd, mm	7.9 ± 0.6	8.2 ± 0.5	11.2 ± 0.9
LWDs, mm	11.6 ± 0.6	13.4 ± 0.6	14.8 ± 0.9
FS, %	30.0 ± 1.2	38.6 ± 1.2	34.0 ± 1.6
EF, %	57.1 ± 2.1	66.2 ± 2.0	63.2 ± 1.5
Presence of segmental left ventricular contractility abnormalities	non	non	non
Presence of abnormal intracardiac flows	non	non	non

**Table 4:** The mean values of QCA measurements at 30 and 90 days post-implantation, where MLD—minimal lumen diameter, RD—reference diameter. Statistical analysis was performed using the unpaired *t* test or the Mann–Whitney test at the statistical significance level of 0.05, depending on the meeting criterion for appropriate test.

	Day 30			Day 90		
	GCS	DES	*p*-Value	GCS	DES	*p*-Value
RD, mm	3.03 ± 0.35	3.05 ± 0.39	0.9244	3.12 ± 0.31	3.23 ± 0.26	0.5354
MLD, mm	2.66 ± 0.41	2.77 ± 0.19	0.2960	2.55 ± 046	2.92 ± 0.25	0.1013
Restenosis, %	12.3 ± 6.1	8.6 ± 5.8	0.2782	18.3 ± 10.5	9.6 ± 6.6	0.1074

**Table 5:** The mean values of OCT measurements at 30 and 90 days post implantation. Statistical analysis was performed using the unpaired *t* test or the Mann–Whitney test at the statistical significance level of 0.05, depending on the meeting criterion for appropriate test.

	Day 30			Day 90		
	GCS	DES	*p*-Value	GCS	DES	*p*-Value
Lumen area, mm^2^	6.49 ± 2.06	7.85 ± 1.65	0.2243	8.52 ± 1.46	9.18 ± 1.26	0.3434
Stent area, mm^2^	8.84 ± 1.08	9.39 ± 1.06	0.3647	10.17 ± 1.22	10.57 ± 1.18	0.5771
Neointimal area, mm^2^	2.35 ± 1.16	1.55 ± 0.78	0.1880	1.65 ± 0.42	1.39 ± 0.59	0.3947
Uncovered struts, %	50%	60%	>0.9999	57%	60%	>0.9999

## Data Availability

The data that support the findings of this study are available from the corresponding author upon reasonable request.

## Abbreviations

The following abbreviations are used in this manuscript:

%DS	percent of diameter stenosis
AG	acute gain
ALT	alanine aminotransferase
Ao	aorta
AST	aspartate aminotransferase
DAPT	dual antiplatelet therapy
DES	drug-eluting stents
ECG	electrocardiogram/electrocardiography
EF	ejection fraction
FS	fractional shortening
GCS	graphene-coated stents
Hb	hemoglobin
HCT	hematocrit
IVSd	interventricular septum in diastole
IVSs	interventricular septum in systole
MLD	minimal lumen diameter
LA	left atrium
LAD	left anterior descending coronary artery
LCx	left circumflex coronary artery
LL	late lumen loss
LVDd	left ventricular diastolic diameter
LVDs	left ventricular systolic diameter
LVEF	left ventricular ejection fraction
LWDd	left ventricular wall in diastole
LWDs	left ventricular wall in systole
OCT	optical coherent tomography
QCA	quantitative coronary angiography
RBC	red blood cell count
RCA	right coronary artery
RD	reference diameter
RVDd	right ventricular diastolic diameter
SEM	scanning electron microscopy
WBC	white blood cell count
